# Secondary Analysis of a Study on Exercise Therapy in Hip Osteoarthritis: Follow-Up Data on Pain and Physical Functioning

**DOI:** 10.3390/ijerph18168366

**Published:** 2021-08-07

**Authors:** Inka Roesel, Benjamin Steinhilber, Peter Martus, Pia Janssen, Inga Krauss

**Affiliations:** 1 Institute for Clinical Epidemiology and Applied Biometry, Medical Faculty, University Hospital Tuebingen, 72076 Tubingen, Germany; inka.roesel@med.uni-tuebingen.de (I.R.); peter.martus@med.uni-tuebingen.de (P.M.); 2 Department of Sports Medicine, Medical Clinic, University Hospital Tuebingen, 72076 Tubingen, Germany; pia.janssen@med.uni-tuebingen.de; 3 Institute of Occupational and Social Medicine and Health Services Research, Medical Faculty, University Hospital Tuebingen, 72076 Tubingen, Germany; benjamin.steinhilber@med.uni-tuebingen.de; 4 Interfaculty Research Institute for Sports and Physical Activity, Tuebingen, 72076 Tubingen, Germany

**Keywords:** hip osteoarthritis, exercise, pain, physical functioning, adherence, patient satisfaction

## Abstract

We evaluated the short- and longer-term effects of exercise therapy in hip osteoarthritis patients (OA) at baseline, three, six, and 12 months in a randomized setting, followed by a non-randomized setting. The primary randomized intervention (E = exercise, P = placebo–ultrasound, C = control) was followed by a voluntary three-month exercise therapy for P and C (renamed P-E, C-E). Participants randomized to E were not offered treatment again (E-C). Effect sizes (ES; 95% CI) were calculated for within-group effects across time for bodily pain (SF-36) and WOMAC pain, function, and stiffness. ANCOVAs of post-treatment scores were used for group comparison after the group-specific exercise intervention phase. Exercise adherence was assessed and related to post-treatment scores of clinical outcomes. Data of 115 participants of the RCT eligible for follow-up and completing exercise therapy were included into our analyses. Small to medium beneficial long-term effects of cumulative interventional effects, including exercise training, persisted in all groups. Group E-C (*n* = 49) showed significant 12 months vs. baseline within-group ES in all outcomes (ES 0.39–0.59) except stiffness. Findings were less prominent for exercise therapy in a non-randomized setting (C-E, P-E, both *n* = 33). Differences are partially explained by adherence rates, highlighting the relevance of therapy compliance strategies. Short-term between-group differences (ANCOVAs) only showed statistically significant differences for WOMAC function between P-E and E-C in favor of E-C (6.4 (95% CI 1.6–11.2; score range 0–100)).

## 1. Introduction

Osteoarthritis (OA) is a degenerative disease that predominantly affects weight-bearing joints, such as the hip and knee. OA ranks among the ten most disabling diseases in high-income countries, and is a major cause of pain, stiffness, and limitation in physical function [1]. Exercise therapy is recommended as the first-line conservative treatment to counteract OA-related symptoms, as highlighted in the guidelines released by the Osteoarthritis Research Society International (OARSI), the American Academy of Orthopedic Surgeons (AAOS), the European League Against Rheumatism (EULAR), and others [2,3,4,5]. Physical exercises have therefore become a common treatment, yet adherence to exercise is critical to achieve long-term sustainable health benefits [6,7], and data suggest a dose relationship between adherence and exercise effects for patients with knee osteoarthritis [8]. Another potential prerequisite for long-time adherence and beneficial outcomes is patient satisfaction. It is an indicator of the efficacy, quality, and feasibility of healthcare services [9].

Several clinical trials have demonstrated the benefits of exercise in patients with knee, hip, or multiple-site OA in the short-term [10,11]. For patients with exclusively hip OA, meta-analyses report small, standardized treatment effect sizes versus control for pain and physical functioning [12,13]. However, there is limited research on this patient group that provides evidence regarding longer-term results (≥6 months) [10,14].

Our present analyses are based on a previously published randomized controlled trial (RCT) on the efficacy of a 12-week exercise program specifically tailored for patients with hip OA. In this trial, participants had been randomly allocated to three groups: exercise therapy (E), placebo ultrasound (P), and the control group (C) receiving no intervention. It had been demonstrated that the exercise therapy was superior to the placebo and control with respect to pain reduction and physical functioning after the three-month intervention phase (η^2^ = 0.42–0.48). Superiority of the exercise group for joint stiffness was minor and statistically non-significant [15,16,17].

This article reports on follow-up data until 12 months (t3 = three months, t6 = six months, t12 = 12 months) after baseline (t0) of the abovementioned RCTs. Patients initially randomized to P or C were asked to participate in the exercise intervention for a consecutive 12-week period after the initial three-month randomized intervention phase (renamed C-E and P-E, respectively); participants of group E did not receive any further treatment throughout the trial (renamed as group E-C). The main objectives of this follow-up study were to assess the course of health outcomes over time in the different sequence groups and to evaluate the effectiveness of hip-specific training in the longer-term. We hypothesized that the three-month exercise training had sustained beneficial effects for all three groups at t12.

A further explorative data analysis investigated if short-term effects differed between the three groups despite receiving the same exercise treatment due to the randomized (E-C, at t3) and non-randomized setting of the intervention (C-E, P-E; both at t6). In addition, the rates of adherence to exercise training were analyzed. Treatment satisfaction and participants’ recommendations of the interventions to others were reported to determine if treatment credibility differed in the different settings of randomized and non-randomized interventions.

## 2. Materials and Methods

### 2.1. Design

A recent RCT, which investigated the efficacy of a three-month intervention period (t0–t3), was followed by a non-randomized voluntary three-month intervention (t3–t6) for participants who had not yet received exercise treatment, and a six-month phasing-out period of no further study treatment (t6–t12). Details on the design and results of the original three-month RCT are presented in separate publications [16,17].

**Figure 1:**
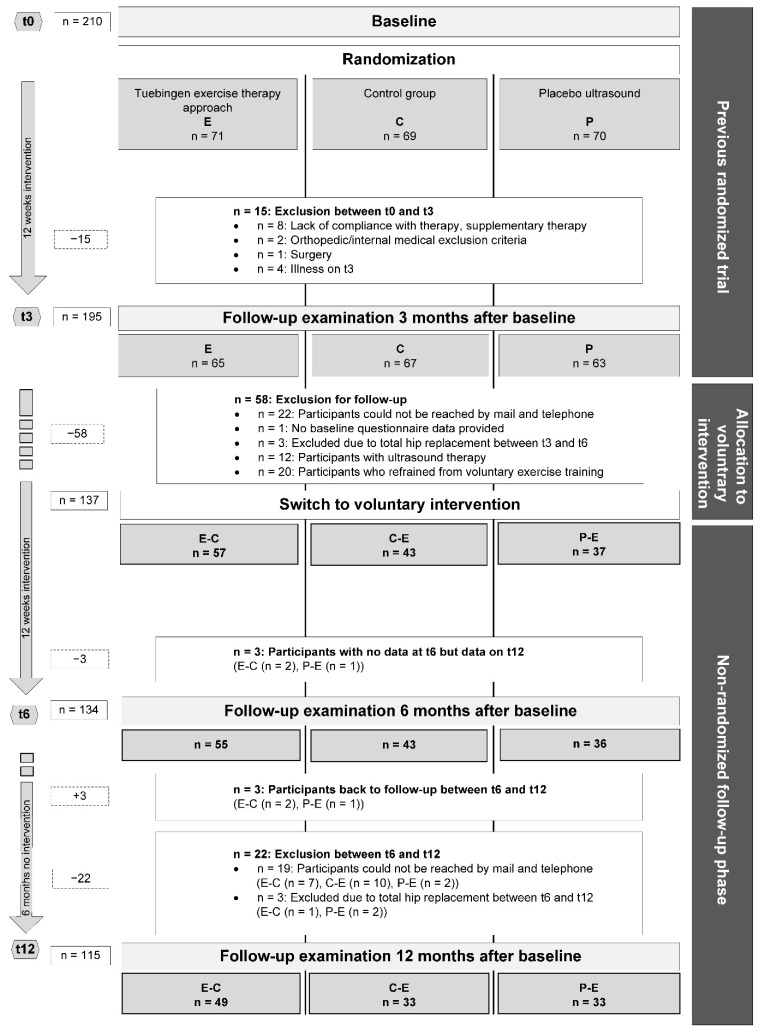
Study flow chart. Tuebingen exercise therapy approach (E); control group (C); placebo ultrasound (P); hyphenated letters indicate group sequences for intervention phases 1 and 2, respectively.

For the primary three-month intervention period between t0 and t3, subjects were randomly assigned to one of the three groups: exercise therapy (E), placebo ultrasound (P), and control (C). As specified in the study protocol of the RCT, subjects who had not yet received exercise therapy (E) in this primary intervention phase were offered to choose exercise therapy for the upcoming three months (t3–t6), similar to a waiting list design [16]. Treatment groups of this follow-up (FU) analysis were named E-C, P-E, and C-E based on their group allocation between t0–t3 and t3–t6, respectively. For details of the treatment allocation and coding of the respective treatment sequence groups, see Figure 1.

In our present article, only patients who were randomized to E at t0 or who opted for the voluntary exercise intervention between t3 and t6 were included into our analysis of this non-randomized, non-controlled follow-up phase, since the number of participants of P and C refraining from the voluntary exercise intervention (and from any other treatment, e.g., ultrasound therapy) was too small to constitute a group. The original RCT phase was reanalyzed with subjects eligible for FU.

### 2.2. Participants

Details on the recruitment of patients with hip OA, as well as general inclusion and exclusion criteria for the original three-month RCT phase, are described in the study protocol and in Supplement 1. To be eligible for FU analysis of this RCT, participants who had completed data assessments at t0 and t3 had to additionally contribute data to at least one of the following time points, t6 or t12, for any of the outcomes of interest. All patients who completed t12 were included in our primary study population of “finishers”. Subjects who fulfilled our eligibility criteria for FU analysis but dropped out before t12 were only included in our sensitivity analysis. For the FU phase analyzed in this paper, further exclusion criteria were specified. Subjects were excluded for the following reasons: hip replacement during the study period, loss to follow-up, and refusal of exercise training or participation in intervention measures other than exercise (see Figure 1).

### 2.3. Interventions

#### 2.3.1. Exercise Therapy (E)

The Tuebingen exercise therapy approach (E) is a 12-week, land-based exercise program specifically designed for patients with hip OA. It comprises supervised group sessions (once a week) and unsupervised home training sessions (twice a week). The program includes exercises to strengthen the muscles of the hip and the lower extremities and to improve balance and flexibility. It further includes exercise and OA-related information to foster health literacy, as well as social interaction within the group sessions. Pain and training were systematically documented in a training log. Further details of the exercise regime are depicted according to the TIDier Checklist [18] in Supplement 2 and elsewhere [16,19]. The exercise regimen E was the same in the primary intervention phase (t0–t3, E-C) as in the voluntary phase (t3–t6, C-E, and P-E, respectively).

#### 2.3.2. Placebo Ultrasound (P)

The placebo ultrasound intervention between t0 and t3 for subjects of group P-E was applied once a week for 15 min by a study assistant. The ultrasound transducer was moved around the hip region with the ultrasound being switched off [16].

#### 2.3.3. Control (C)

Participants initially randomized to the control group did not receive any study intervention in the primary intervention phase (t0–t3, C-E). After t6, none of the groups received any further treatment.

### 2.4. Measures

#### 2.4.1. Primary Outcome

In line with the previously published randomized controlled trial for the initial intervention phase between t0 and t3, the primary outcome measure was the bodily pain subscale of the 36-item Short Form (SF-36). The SF-36 is a widely used self-completed questionnaire consisting of 36 items recording eight dimensions of subjective health. Each of the eight subscales can be used independently. Scores range from 0 to 100, with higher values indicating better health. Internal consistency for the subscale of bodily pain is specified with Cronbach’s α = 0.76 for a German population with chronic lumbar back pain and α = 0.88 for the German norm population of 1996 [20]. The SF-36 captures improvements in pain in hip and knee osteoarthritis patients undergoing inpatient rehabilitation intervention with a focus on active physical therapy, however, functional improvement can better be captured by the WOMAC Index [21]. Further information on convergent validity is outlined below.

#### 2.4.2. Secondary Outcomes

The Western Ontario McMaster Universities Osteoarthritis Index (WOMAC^®^ NRS for Germany 3.1 Index) was used to evaluate OA-related hip pain, physical functioning, and stiffness. The WOMAC is a self-administered health status instrument consisting of 24 questions divided into three subscales: pain (five items), physical function (17 items), and joint stiffness (two items). Each subscale was transformed to a score from 0 (no impairment) to 100 (worst impairment). Among patients with hip OA, the WOMAC has demonstrated an excellent test–retest reliability (ICC 0.77–0.94) and a good to excellent construct validity with SF-36 bodily pain (r = 0.64–0.67) and SF-36 physical function (r = 0.73) [22,23]. Improvement in pain and function in hip and knee OA rehabilitation patients can both be captured with the WOMAC Index [21].

Adherence to group-based exercise sessions (reported by the supervisor) and home-based exercise sessions (self-report) was tracked during the intervention periods and assessed by the percentage of attended versus all scheduled sessions.

A five-point Likert Scale was used at t3 and t6 to quantify satisfaction with the treatment and to investigate whether participants would recommend this therapy to others. Anchors of the Likert scale were ‘applicable’ (1), ‘likely applicable’, ‘neither nor’, ‘likely inapplicable’, ‘inapplicable’ (5).

#### 2.4.3. Confounders

Age, sex, body mass index (BMI), and educational level were recorded to characterize patients at baseline t0.

### 2.5. Sample Size

No separate sample size calculation was conducted for this secondary analysis. Sample size calculation was only related to the primary endpoint of the initial three-month RCT trial phase, which was the change of the SF-36 subscale bodily pain from t0 to t3 of group E in comparison to group C. Details are provided in the study protocol [16].

For group E-C with a sample size of *n* = 49, a mean of paired differences of 7.1 between t12 and t0 for bodily pain was detected with a power of 80% and a standard deviation of paired differences of 17.4 (t12 vs. t0), assuming a type I error of 0.05 (two-sided) (nQuery version 8.4.1.0, Statsols, San Diego, CA, USA).

### 2.6. Statistical Analysis

With the three sequence groups E-C, P-E, and C-E, an analysis of patients participating across the entire follow-up period (finishers) was performed. As we are aware of a potentially reduced precision of estimates caused by the problem of attrition, a sensitivity analysis including non-finishers and finishers and a comparison of baseline data of both samples were performed to examine whether our results showed any selection bias as a consequence of systematic dropouts between t6 and t12.

Distributions were assessed based on histograms and their skewness and kurtosis. Group comparisons at baseline (t0) were performed using one-way analysis of variance (ANOVA) for continuous variables and a chi-squared test, as well as a Fisher–Freeman–Halton test for categorical variables.

Descriptive methods (mean, standard deviation (SD), change scores from baseline) were used to depict the course of the SF-36 subscale of bodily pain, as well as the WOMAC subscales of pain, physical functioning, and stiffness over time for each sequence group separately. Negative change scores for WOMAC subscales and positive change scores for SF-36 indicated improvement. In addition, effect sizes (ES) and 95% confidence intervals (CI) of mean differences between each follow-up time point (t3, t6, t12) and baseline (t0), as well as for t6 and t12 versus t3 (exercise intervention period and follow-up period for P-E and C-E), were calculated. The mean differences were approximately normally distributed. The effect sizes were estimated using Hedges’ g_z_ with Cohen’s d_z_ for correlated within-subject observations and a bias correction factor to account for small sample sizes [24]. Positive values indicate benefit. The interpretation of effect sizes referred to the threshold values of 0.2 for small effects, 0.5 for medium effects, and 0.8 for large effects [25]. Additionally, we performed paired *t*-tests to examine the statistical significance of each change score separately. The null hypothesis was that there was no change from baseline.

To compare the effect of the exercise intervention between the three groups E-C, P-E, and C-E in view of contextual differences of the intervention between t0–t3 and t3–t6, analysis of covariance (ANCOVA) of post-treatment scores directly after exercise therapy (t3 for E-C and t6 for P-E and C-E) adjusted for pre-treatment values (t0 for E-C and t3 for P-E and C-E) was performed for each health outcome. To account for violations of the normal distribution assumption, the Kruskal–Wallis test was applied to assess differences in adherence to training between the different groups. Since adherence was not independent of group effects and homogeneity of regression slopes was violated, partial Spearman’s rank correlations were used to explore the relation between adherence and post-treatment scores of clinical outcomes (controlling for pre-treatment scores). A significance level of α = 0.05 (two-sided) was set for all statistical inferences. All analyses were conducted with R (*R* version 3.6.1 and RStudio 1.1.383, RStudio, PBC, Boston, MA, USA) and SPSS (Version 26, IBM Corp, Armonk, NY, USA).

## 3. Results

### 3.1. Participant Flow

The 210 subjects included in the original RCT [17] were randomized to one of the three modalities E, C, and P at baseline (t0). A total of 137 patients provided data at t0, t3, t6, and/or t12 and participated in exercise training between t0–t3 or t3–t6, and were thus eligible for our FU analysis. At t3, 61% of the 130 patients randomized to C (*n* = 67) or P (*n* = 63) for the first three months opted for E as voluntary treatment in the second intervention phase (C-E, *n* = 43 (64.1%); P-E, *n* = 37 (58.7%)). Ultimately, 115 participants completed t12 and were included into the finisher analysis (E-C = 49, C-E = 33, and P-E = 33). The sensitivity analysis related to *n* = 137. Further details on group sizes and numbers of participants lost to follow-up are depicted in Figure 1.

### 3.2. Participant Characteristics

The baseline characteristics of the participants of E-C, C-E, and P-E are presented in Table 1 (*n* = 115). Patients ranged from 34 to 80 years of age, and 30–42% of the participants in each group were female. It is worth mentioning that over 95% of participants in each group had a higher vocational education or university degree. No statistically significant differences between subjects of the three treatment sequences were observed for any of these baseline measures.

### 3.3. Primary Objective: Long-Term Responsiveness (t0-t12) within Sequence Groups

Mean values (SD), change scores, and effect sizes (95% CI) between follow-up time points versus t0 for clinical outcomes are shown in Table 2 and Supplement 3.

In group E-C, the SF-36 bodily pain, as well as WOMAC pain, physical functioning, and stiffness scores significantly improved during the first three months of exercise training (t0–t3). This benefit was preserved until the end of the study in all outcomes except for WOMAC stiffness. According to our hypotheses, effect sizes for the SF-36, WOMAC pain and physical functioning, and time windows between t3, t6, and t12 versus t0 were small to medium and statistically significant (WOMAC pain and physical functioning ES 0.50 to 0.63, SF-36 bodily pain ES 0.31 to 0.39, 95% CI did not include 0) in this group. For WOMAC stiffness, significant effects were preserved until t6, however, these did not reach statistical significance at t12 vs. baseline (ES 0.26, 95% CI (−0.03, 0.54)).

C-E did not improve in any measure between t0 and t3, but benefited significantly throughout the exercise intervention period (t3–t6) with respect to all measures, except WOMAC stiffness; SF-36 bodily pain (ES 0.50, 95% CI (0.13, 0.86)) and WOMAC pain (ES 0.38, 95% CI (0.03, 0.74)) decreased, and WOMAC physical functioning (ES 0.40, 95% CI (0.05, 0.76)) improved (Table 3). Subjects of group C-E showed moderate sustained improvements six months after ceasing the exercise intervention (t0–t12) for the subscales WOMAC pain, physical functioning, and stiffness.

Group P-E experienced moderate statistically significant changes after the placebo ultrasound treatment (t0–t3) in the WOMAC subscales of pain (ES 0.37, 95% CI (0.02, 0.73)), physical functioning (ES 0.49, 95% CI (0.12, 0.84)), and stiffness (ES 0.49, 95% CI [0.13, 0.84)), however, not in the primary outcome of SF-36, bodily pain. Exercise training in the voluntary setting for group P-E (t3–t6) after the primary placebo intervention also had a significant effect on the reduction of pain (WOMAC pain ES 0.37, 95% CI (0.01, 0.73)) (see Table 3). Longer-term statistically significant improvements at t12 were preserved for WOMAC pain (ES 0.33. 95% CI (0.02, 0.68)) and stiffness (ES 0.49, 95% CI (0.12, 0.84)) (Table 2), however, excluding placebo effects from the primary intervention phase, no sustained significant beneficial effects could be observed at t12 in any of the outcome measures (Table 3; t3–t12).

### 3.4. Explorative Analysis: Comparisons of Sequence Groups

Pre-exercise treatment baseline values (t0 for E-C; t3 for C-E and P-E) did not differ significantly for any of the health outcomes (SF-36, bodily pain *p* = 0.76; WOMAC pain *p* = 0.41; WOMAC physical functioning *p* = 0.54; WOMAC stiffness *p* = 0.74). To investigate sequence group differences with respect to the efficacy of the exercise intervention in a randomized versus non-randomized setting, ANCOVAs on post-treatment scores (t3 for E-C and t6 for C-E and P-E) controlling for pre-treatment scores (t0 for E-C and t3 for C-E and P-E) were conducted for each health outcome separately (Table 4). No results of statistical significance were found, except for WOMAC function (F (2,110) = 3.52; *p* = 0.03). The baseline-adjusted mean difference between sequence groups P-E and E-C was found to be 6.41 (95% CI (1.61, 11.22); *p* = 0.009); the baseline-adjusted mean difference between sequence groups C-E and E-C (2.97; 95% CI (−1.78, 7.72)), however, was not significant (*p* = 0.22).

### 3.5. Adherence to Exercise

The Kruskal–Wallis test results showed significant differences in the adherence to exercise between the three sequence groups (*p* < 0.001). Median adherence to exercise in group E-C (97.2%) was significantly higher than in groups C-E (median 85.3%; *p* < 0.001) and P-E (median 85.3%; *p* < 0.001). No significant difference could be found between groups C-E and P-E (*p* = 0.90). Besides these findings, it is worth mentioning that within-group variances differed strongly between the three groups, with high interindividual variability in groups C-E and P-E (see Table 5).

Partial Spearmen’s rank correlations were used to explore the relation between adherence and the post-treatment scores of all clinical outcomes controlling for baseline measures. There was a moderate negative correlation between adherence and WOMAC pain post-treatment score while controlling for pre-treatment WOMAC pain scores (rho = −0.327; *n* = 114; *p* < 0.001). The same applied for the relationship between adherence and WOMAC functioning post-treatment scores with a negative correlation of rho = −0.303 (*n* = 114; *p* = 0.001) controlling for pre-treatment WOMAC functioning score and for WOMAC stiffness post-treatment scores with a negative correlation of rho = −0.271 (*n* = 114, *p* = 0.004) controlling for pre-treatment scores. No significant correlation between adherence and SF-36 bodily pain post-treatment scores controlling for pre-treatment scores could be found (rho = 0.104; *p* = 0.272).

### 3.6. Treatment Satisfaction

Almost all of the 115 patients analyzed provided answers about their satisfaction with the exercise therapy regime (*n* = 112, 97.4%). The majority of participants (*n* = 106, 94.6%) was satisfied (‘applicable’; *n* = 94 (83.9%) or ‘likely applicable’; *n* = 12 (10.7%)) with the exercise intervention and would recommend it to others (*n* = 109, 97.3%). Details on the numbers for each sequence group are depicted in Supplement 4.

### 3.7. Sensitivity Analysis

The results of the sensitivity analysis are depicted in Supplements 5–7. On average, finishers frequently responded better to treatment. Some effects were only statistically significant for the sample of finishers. This held true for WOMAC pain in period t0–t3 (P-E), as well as SF-36 pain reduction (E-C) and WOMAC pain (C-E) in period t0–t6 (numbers above, Table 2 and Supplement 5).

## 4. Discussion

This study presents a secondary analysis of non-controlled follow-up data of a previously published randomized controlled trial on the short-term effects of exercise therapy in hip OA. The first objective was to examine the sustainment of intervention effects throughout the follow-up period. We expected beneficial effects up to 12 months of follow-up. Hip OA patients who were initially randomized into a three-month, hip-specific exercise program and received no further treatment until the end of the study (E-C) showed small to moderate effects on pain reduction (WOMAC, SF-36) and physical function (WOMAC) even nine months after cessation of the supervised training period. We also investigated health outcomes in two groups that were initially randomized to control (C-E) or placebo–ultrasound (P-E) for three months and subsequently opted for the exercise intervention for the following three months, according to a waiting list-like design. Participants of those two sequence groups showed sustained small to medium reductions in WOMAC pain and stiffness (P-E, C-E) and improvements in physical functioning (C-E) at the end of the study (t12), however, not for SF-36, bodily pain. It further has to be noticed that P-E did not show any relevant effect for the SF-36 subscales of bodily pain, physical functioning, and stiffness between t3 and t6. The results of the sensitivity analysis demonstrated even smaller effect sizes. Isolating the voluntary exercise intervention from the placebo intervention, longer-term beneficial effects could not be sustained (t12 versus t3).

The results of this trial can provide important information to support existing knowledge on this issue: Sampath et al. recently conducted a meta-analysis indicating small to moderate long-term effects of exercise in comparison to the control in patients with hip OA [12]. The analysis included data of about 500 subjects with follow-up data of three to twelve months after baseline. The underlying exercise programs are comparable in many ways to our exercise intervention (Supplement 2): three of the studies cited offered at least one weekly supervised exercise session over a period of three months [14,27,28] (with a minimum of 6–8 supervised sessions [29,30]); all studies included self-administered training in the gym or at home on top of the supervised weekly training sessions, and all of the studies focused on strengthening exercises [14,27,28,29,30,31]. In addition, all but one mentioned flexibility training to be included in the training program [14,27,28,29,31], and two studies explicitly state the integration of functional or proprioceptive [27,31] exercises; four studies expressly declared that they had used an exercise program specifically designed for patients with hip OA [14,27,29,30]. Although individual study-level results mostly failed to demonstrate statistically significant prolonged exercise effects, meta-analysis data [12] revealed a small overall effect on long-term pain reduction and a moderate effect on physical functioning, with standard mean differences of 0.24 (95% CI (0.41, 0.06)) and 0.33 (95% CI (0.15, 0.50)), respectively. These long-term effects are smaller than those reported in our follow-up study. This is consistent with the short-term intervention effects, which were superior in our analysis as well (Table 2).

Our follow-up analysis did not compare outcome measures versus a control group. It further focused on finishers willing to participate until the end of the study. Both facts may have an impact on effect sizes. However, for group E-C, our sensitivity analysis with the data of both finishers and non-finishers combined (*n* = 57) is quite similar to the data of finishers alone (*n* = 49, Supplement 5, Table 2). The lack of a control group will be addressed in the limitations section below.

Randomized controlled trials provide ideal preconditions to prove efficacy, and therefore may induce an overestimation of benefits [32]. The primary intervention phase represents a randomized controlled trial design, whereas the second voluntary phase with the option of patients to opt for or refrain from the intervention may have increased the character of a real-world scenario. Although the health professional instructing the exercise instructor was obliged to guide the intervention identically to the randomized phase, a performance bias cannot completely be ruled out. Moreover, subjects may have been less satisfied with the treatment. Yet, this argument is not clearly underpinned when looking at the reported satisfaction with the exercise intervention and its recommendation to others. Both satisfaction and recommendations were most prominent in group E-C, but exercise treatment evaluations of the two other groups (C-E, P-E) showed only slightly smaller satisfaction and willingness to recommend treatment to others than E-C. This difference does not seem to explain the obvious differences in outcome effects.

Whereas the preceding discussion refers to potential context effects of the intervention, adherence to exercise may directly influence the inherent effects of exercise. One possible explanation for differences in treatment effects is related to adherence rates to group and home exercises. Exercise adherence rates were statistically significantly smaller for the two waiting list-like groups, P-E and C-E, during the voluntary intervention phase in comparison to adherence rates of group E-C during the randomized treatment period. It is possible that the higher adherence rates in the E-C group are due to the so-called Hawthorne effect, which is described as the awareness of being involved in a trial that can result in an altered (i.e., improved) behavior or performance [33]. Participants who did not receive the exercise program until after the first RCT phase might have lacked motivation or lost interest during the intervention, not feeling obliged to adhere. Treatment effects for WOMAC pain, physical functioning, and stiffness were inversely correlated to adherence rates. These results may explain the differences in treatment effects in the two different exercise intervention periods, as it is well-known from other studies that adherence to exercise correlates with effectiveness [6,34].

When interpreting the results, it has to be kept in mind that the effects at t12 may not only be a cumulative result of the preceding follow-up periods t0–t3, t3–t6, and t6–t12, but it may further be a consequence of an attrition bias due to systematic loss to follow-up. A disproportionate study dropout rate of subjects who did not respond to the intervention may affect the data that are used for the description of long-term effects. In this respect, the results of C-E are most prone to this potential bias, as almost a quarter of its subjects were lost to follow-up from t6 (*n* = 43) to t12 (*n* = 33). Finishers in group C-E responded better to the treatment between t3 and t6, which is indicated by the statistically significant small to medium effect sizes for WOMAC pain and physical functioning in comparison to the corresponding smaller and non-significant effect sizes in the sensitivity analysis (Table 3 and Supplement 6). Sensitivity analysis of P-E showed comparable values for exercise treatment effects for WOMAC pain. The data of P-E are influenced by two subjects who dropped out due to joint replacement between t6 and t12. Individual data of these patients indicate a clinically relevant worsening of symptoms at t6 prior to surgery. These data negatively affect the effect sizes of the exercise intervention period between t3 and t6 for the sensitivity sample as well, and underpin the presence of an attrition bias in the dataset of finishers.

### Limitations

The education level of the sample under study was not representative, as almost every study participant had a higher education, and more than one-third of each group held a university degree [35]. According to the fact that a higher educational level is correlated with better health behavior [36], the transferability of the results of this study including a large number of participants with a high educational level to populations with a minor educational standard is limited.

It also remains a methodological drawback of this study that follow-up data could no longer be compared to a control group. Patients randomized to the control or placebo ultrasound for the first study period between t0 and t3 were subsequently free to participate in the exercise intervention. This design was chosen for reasons of compliance, as all participants should have had the possibility of access to exercise therapy within a short period of time. As a consequence, the number of patients not participating in exercise at any time throughout the study was too small to add up to sufficiently large control groups. Furthermore, the study period subsequent to t3 was neither randomized nor controlled, and potential confounders, as well as other external factors that directly influence outcomes, may partially explain the observed effects as well. Lastly, in group P-E, the cumulative effects of the primary placebo intervention and following exercise treatment cannot be completely separated.

## 5. Conclusions

This explorative analysis emphasizes the benefit of exercise interventions in hip OA in the long-term for patients characterized by a moderate disease state and a high level of education. However, it cannot be ruled out that the treatment effects of a randomized controlled trial setting may overestimate the true treatment effects in general healthcare. Furthermore, adherence to exercise therapy might be higher in RCTs than in everyday routines. Pragmatic trials in a regular health service setting are therefore highly recommended to produce results with a high external validity that can be generalized and applied in routine settings [32,37].

## Figures and Tables

**Table 1:** Baseline characteristics of all finishers (*n* = 115).

	Total (*n* = 115)	E-C (*n* = 49)	C-E (*n* = 33)	P-E (*n* = 33)	*p*
**Age (years)** (*n* = 115)					0.59
mean (SD)	57 (9.6)	57 (10.1)	59 (9.4)	56 (9.2)	
**Gender** (*n* = 115)					0.47
female; *n* (%)	39 (33.9)	15 (30.6)	10 (30.3)	14 (42.4)	
male	76 (66.1)	34 (69.4)	23 (69.7)	19 (57.6)	
**BMI** (*n* = 115)					0.42
mean (SD)	26.5 (3.4)	26.7 (3.6)	26.9 (3.4)	25.9 (3.3)	
**Education** (*n* = 111)					0.25
university; *n* (%)	50 (45.0)	23 (47.0)	17 (56.7)	10 (31.3)	
higher vocational education	58 (52.3)	25 (51.0)	12 (40.0)	21 (65.6)	
basic secondary school	3 (2.7)	1 (2.0)	1 (3.3)	1 (3.1)	
**SF-36, bodily pain** (*n* = 115)					0.52
mean (SD)	58.1 (18.0)	60.3 (16.6)	57.1 (19.0)	55.9 (19.2)	
**WOMAC, pain** (*n* = 115)					0.52
mean (SD)	27.0 (16.2)	25.0 (14.4)	29.0 (18.3)	27.8 (16.6)	
**WOMAC, physical functioning** (*n* = 115)					0.50
mean (SD)	26.0 (15.9)	24.0 (15.6)	27.4 (17.2)	27.6 (15.3)	
**WOMAC, stiffness** (*n* = 115)					0.17
mean (SD)	33.7 (21.4)	29.4 (18.5)	36.4 (24.9)	37.6 (21.3)	

**Table 2:** Mean (SD), change scores = change from baseline within-group means, effect sizes (Hedges g_z_ [26]), and 95% CIs for clinical outcome measures of finishers (*n* = 115).

	E-C (*n* = 49)		C-E (*n* = 33)		P-E (*n* = 33)	
	Mean (SD)	Change Score (ES; 95% CI)	Mean (SD)	Change Score (ES; 95% CI)	Mean (SD)	Change Score (ES; 95% CI)
**SF-36 bodily pain**						
**t0**	60.3 (16.6)		57.3 (19.3)		55.8 (19.2)	
**t3**	67.1 (18.8)	6.80 * (**0.36**; (0.07; 0.65))	55.7 (19.1)	−1.63 (−0.10; (−0.44, 0.25))	57.5 (18.9)	1.64 (0.08; (−0.26, 0.42))
**t6**	66.8 (21.2)	6.35 * (**0.31**; (0.02, 0.61))	64.6 (20.0)	7.25 (0.34; (−0.01, 0.70))	61.1 (17.2)	5.37 (0.35; (−0.01, 0.71))
**t12**	67.1 (19.2)	6.86 * (**0.39**; (0.10, 0.68))	60.3 (22.0)	3.03 (0.15; (−0.20, 0.49))	60.1 (20.1)	4.21 (0.21; (−0.14, 0.55))
**WOMAC pain**						
**t0**	25.0 (14.4)		29.0 (18.3)		27.8 (16.4)	
**t3**	16.9 (15.0)	−8.16 * (**0.57**; (0.27, 0.87))	29.2 (19.8)	0.12 (−0.01; (−0.34, 0.33))	22.4 (14.2)	−5.39 * (**0.37**; (0.02, 0.73))
**t6**	17.3 (15.6)	−8.17 *(**0.50**; (0.19, 0.80))	22.3 (17.7)	−6.73 * (**0.37**; (0.02, 0.73))	17.9 (13.1)	−10.37 * (**0.65**; (0.27, 1.03))
**t12**	16.9 (13.4)	−8.08 * (**0.56**; (0.25, 0.86))	21.9 (15.7)	−7.09 * (**0.37**; (0.02, 0.72))	22.3 (15.7)	−5.52 (**0.33**; (0.02, 0.68))
**WOMAC function**						
**t0**	24.0 (15.6)		27.4 (17.2)		27.6 (15.1)	
**t3**	15.6 (14.1)	−8.44 * (**0.63**; (0.32, 0.93))	25.6 (15.9)	−1.86 (0.14; (−0.20, 0.48))	21.0 (13.0)	−6.58 * (**0.49**; (0.12, 0.84))
**t6**	15.9 (15.7)	−8.38 * (**0.50**; (0.20, 0.81))	19.4 (13.9)	−8.06 * (**0.52**; (0.16, 0.88))	20.4 (12.3)	−7.32 * (**0.54**; (0.17, 0.91))
**t12**	14.9 (13.8)	−9.03 * (**0.59**; (0.28, 0.89))	20.6 (14.0)	−6.86 * (**0.47**; (0.11, 0.83))	22.6 (15.4)	−4.98 (0.32; (−0.03, 0.67))
**WOMAC stiffness**						
**t0**	29.4 (18.5)		36.4 (24.9)		37.6 (21.3)	
**t3**	20.4 (18.2)	−8.98 * (**0.43**; (0.13, 0.72))	27.1 (20.5)	−9.24 * (**0.49**; (0.13, 0.86))	27.7 (16.3)	−9.85 * (**0.49**; (0.13, 0.84))
**t6**	22.4 (17.8)	−7.23 * (**0.33**; (0.03, 0.62))	25.0 (17.4)	−11.36 * (**0.65**; (0.27,1.02))	26.9 (15.4)	−11.25 * (**0.53**; (0.16, 0.90))
**t12**	24.1 (17.9)	−5.31 (0.26; (−0.03, 0.54))	23.0 (16.0)	−13.33 * (**0.55**; (0.18, 0.91))	28.5 (18.1)	−9.09 * (**0.49**; (0.12, 0.84))

ES in bold indicates that the CI does not contain 0. * *p* < 0.05. Null hypothesis: difference (mean at follow-up minus mean at baseline) = 0. The equation for the CI of the ES is not equivalent to the paired *t*-test. Therefore, in one case, the paired *t*-test was not significant, although the CI did not span 0.

**Table 3:** Effect sizes (Hedges g_z_ [26]) and 95% CIs for clinical outcome measures (*n* = 115) in the voluntary exercise intervention period (t3–t6) considering t3 as baseline (P-E and C-E).

	Exercise Intervention Period For P-E, C-E (ES, 95% CI)	Follow-Up (ES, 95% CI)
**C-E (*n* = 33)**	**t3** **–t6**	**t3** **–t12**
SF-36 bodily pain	0.50 (0.13, 0.86)	0.22 (−0.13, 0.57)
WOMAC pain	0.38 (0.03, 0.74)	0.38 (0.03, 0.74)
WOMAC function	0.40 (0.05, 0.76)	0.34 (0.01, 0.69)
WOMAC stiffness	0.13 (−0.21, 0.46)	0.25 (−0.09, 0.59)
**P-E (*n* = 33)**	**t3** **–t6**	**t3** **–t12**
SF-36 bodily pain	0.27 (−0.08, 0.62)	0.12 (−0.22, 0.46)
WOMAC pain	0.37 (0.01, 0.73)	0.01 (−0.33, 0.35)
WOMAC function	0.10 (−0.24, 0.44)	−0.15 (−0.50, 0.19)
WOMAC stiffness	0.09 (−0.26, 0.42)	−0.05 (−0.39, 0.28)

Positive effect sizes (ES) indicate clinical benefit. CI = confidence interval. ES in bold indicates that the CI did not span 0.

**Table 4:** ANCOVA for exercise intervention phase adjusted for baseline (*n* = 115).

	*p*	E-C (*n* = 49)	C-E (*n* = 33)	P-E (*n* = 33)	Difference C-E and E-C **	*p*	Difference P-E and E-C **	*p*
**SF-36 bodily pain**								
pre (mean, SD)		60.3 (16.6)	55.7 (19.1)	57.5 (18.9)				
post		67.1 (18.8)	64.6 (20.0)	61.1 (17.2)				
ANCOVA	0.489	65.8 * (2.7)	65.7 * (2.25)	61.8 * (2.8)	0.11 (−7.17; 6.96)	0.977	−3.98 (−11.10; 3.13)	0.270
**WOMAC pain**								
pre (mean, SD)		25.0 (14.4)	29.2 (19.8)	22.4 (14.2)				
post		16.9 (15.0)	22.3 (17.7)	17.9 (13.1)				
ANCOVA	0.488	17.2 * (1.8)	20.5 * (2.2)	19.2 * (2.25)	3.35 (−2.34; 9.05)	0.246	2.07 (−3.65; 7.79)	0.475
**WOMAC function**								
pre (mean, SD)		24.0 (15.6)	25.6 (15.9)	21.1 (13.0)				
post		15.5 (14.1)	19.4 (13.9)	20.4 (12.3)				
ANCOVA	0.033	15.4 * (1.5)	18.3 * (1.85)	21.8 * (1.89)	2.97 (−1.78; 7.73)	0.218	6.41 (1.61; 11.22)	0.009
**WOMAC stiffness**								
pre (mean, SD)		29.4 (18.5)	27.1 (20.5)	27.7 (16.3)				
post		20.4 (18.2)	25.0 (17.5)	26.9 (15.4)				
ANCOVA	0.084	19.9 * (2.2)	25.6 (2.6)	27.0 (2.7)	5.63 (−2.43; 13.69)	0.225	7.05 (−1.08; 15.17)	0.103

* Adjusted means (standard error); ** difference between adjusted means (95% CI). Post hoc tests adjusted for multiple testing (Bonferroni). Bold font indicates statistical significance.

**Table 5:** Adherence to exercise training in % (*n* = 115).

	E-C (*n* = 49) t0–t3		C-E (*n* = 33) t3–t6		P-E (*n* = 33) t3–t6	
	Mean (SD)	Median (IQR)	Mean (SD)	Median (IQR)	Mean (SD)	Median (IQR)
Group Sessions (GS)	93.7 (7.9)	100 (8.3)	80.0 (21.1)	90.0 (25.8)	78.1 (23.9)	83.3 (21.6)
Home Sessions (HS)	97.6 (5.0)	100 (2.1)	76.6 (26.4)	87.5 (27.1)	69.4 (33.2)	83.3 (58.3)
Total (GS + HS)	96.3 (4.4)	97.2 (8.33)	77.9 (22.5)	85.3 (20.6)	72.3 (28.7)	85.3 (45.7)

Kruskal–Wallis: E-C vs. C-E *p* < 0.001; E-C vs. P-E *p* < 0.001; C-E vs. P-E *p* = 0.90.

## Data Availability

Most data generated or analyzed in this study are included in this published article and its supplementary files. Further datasets used during the current study are available from the corresponding author on reasonable request.

## Supplementary Materials

The following are available online at https://www.mdpi.com/article/10.3390/ijerph18168366/s1, Supplement 1: Inclusion and exclusion criteria for the study, Supplement 2: TIDieR_Amendment, Supplement 3: Forest Plots showing Hedges g_z_ and 95%-CIs for C-E, P-E, E-C for all follow-up time-points versus baseline, Supplement 4: Treatment satisfaction and recommendation (*n* = 112), Supplement 5: Mean (SD), change scores$, effect sizes (Hedges g_z_ 12) and 95%-CIs for clinical outcome measures (*n* = 137), Supplement 6: Effect sizes (Hedges g_z_ 13) and 95%-CIs for clinical outcome measures (*n* = 137) in the exercise intervention period, Supplement 7: ANCOVA for exercise intervention phase adjusted for baseline (*n* = 137).
