# Supplementary material for: Secondary Analysis of a Study on Exercise Therapy in Hip Osteoarthritis: Follow-Up Data on Pain and Physical Functioning
Source: Int J Environ Res Public Health. 2021 Aug 7;18(16):8366. doi: 10.3390/ijerph18168366 (PMC8393441; doi:10.3390/ijerph18168366)
Supplement: Supplementary file 1 [file ijerph-18-08366-s001.zip › ijerph-1279695-supplementary/ijerph-1279695-supplementary final/Roesel_Supplement_1_in_exclusion2021.pdf]

Supplement 1: Inclusion and exclusion criteria for the study

Inclusion criteria

- Osteoarthritis (OA) of one or both hip joint(s)
- Age between 18 and 85 years
- The subject is physically fit for the intervention measure (as ascertained during the examination conducted by the principal investigator). "Fitness" in this setting relates to the physical as well as the psychological condition of the subject. (Subjects will not be excluded if they have one hip endoprosthesis, as long as the contralateral hip is affected by osteoarthritis according to the listed criteria.)
- The subject has the time available to undertake the interventions and attend the measurements
- The subject voluntarily consents to study participation after receiving oral and written information about study content and objectives

Exclusion criteria

- Unstable anchoring in case of total hip replacement at the contra-lateral joint, if applying to the subject.
- Hip dislocation after total hip replacement at the contra-lateral joint, if applying to the subject.
- Further disorders affecting the lower extremities or lower back that require treatment by a physician/therapist and which are not related to OA and are currently being treated.
- Previous trauma at the hip or pelvis area with accompanying development of secondary OA.
- Known endocrinological causes of hip OA.
- Confirmed metabolic causes of hip OA
- State after aseptic bone necrosis (Perthes' disease).
- Presence of OA in several joints (for example, hip and knee) is NOT an exclusion criterion.
- Cardiocirculatory disorders or other comorbidities that result in severely restricted everyday physical capacity and that are contraindications to physical exertion (for example, heart failure NYHA III–IV, terminal renal failure stage IV).
- Medical exercise therapy, physiotherapy on resistance machines in the preceding 3 months, with a total treatment frequency of more than 6 units.
- Systematic group or individual therapy to treat the osteoarthritis (systematic in the sense of a minimum of 1x/week for 30 minutes or more) in the preceding 3 months.
- Physical therapy to treat the osteoarthritis (systematic in the sense of regular, prescribed application at least 1x/week) in the preceding 3 months.
- Newly initiated exercise/movement therapy in the preceding 3 months (sports and movement therapy defined as taking place a minimum of 1x/week, getting out of breath, minimum duration 30 minutes).
- Corticosteroid injection into the hip joint in the preceding 12 months.
- Medication or alcohol misuse.
- Acute illness.
- Use of walking aids.
- Participation in a clinical study in the preceding 4 weeks.
- Lack of compliance.
- Lack of capacity to consent.
