# Supplementary material for: Secondary Analysis of a Study on Exercise Therapy in Hip Osteoarthritis: Follow-Up Data on Pain and Physical Functioning
Source: Int J Environ Res Public Health. 2021 Aug 7;18(16):8366. doi: 10.3390/ijerph18168366 (PMC8393441; doi:10.3390/ijerph18168366)
Supplement: Supplementary file 1 [file ijerph-18-08366-s001.zip › ijerph-1279695-supplementary/ijerph-1279695-supplementary final/Roesel_Supplement_2_TIDieR_Amendment_2021.pdf]

1. Brief name (see main document pp. 1, 3, 4)

2. Why (see main document p. 4)

3. What Materials:

- Elastic rubber bands, stability trainers (pads), exercise balls and exercise mats
- Training logs (documentation of home training sessions (exercises with reps, sets, pain, BORG)
- Information materials on: Pain scale, anatomy, leg alignment etc.

4./8. What Procedures and **When and How much**

- Hip specific elements of motor learning and mobilization, strength training and exercises to improve postural control. Group sessions further include education related to exercises, such as information on anatomical basics and training modalities. The sessions enhance social contacts by having group-based introductions and feedbacks before and after the exercises, and by enforcing partner and group exercises. In the group lessons, subjects are introduced to the exercises they have to do at home.
- Provision of a detailed description (text and image) of all home exercises and their dosage (frequency, intensity, time (duration) and type). The description of exercises and theoretical contents of the upcoming training week were handed out to the participants at the beginning of each week.
- Training progression for strength exercises:

Week	Objective	Intensity (% max strength)	Repetitions / Sets
**1-3**	Motor learning	< 30 %	≥ 30 / 1
**4-8**	Strength endurance	30-40 %	20-25 / 2-3
**9-12**	Endurance & maximum strength	40-70 %	10-15 / 3-4

<u>Sources where further information can be accessed:</u>

- THueKo Intervention description German. DOI: 10.13140/RG.2.2.17966.87363
- Krauss I, Steinhilber B, Haupt G, Miller R, Grau S, Janssen P. Efficacy of conservative treatment regimens for hip osteoarthritis--evaluation of the therapeutic exercise regime "Hip School": a protocol for a randomised, controlled trial. BMC Musculoskelet Disord. 2011;12: 275 and figures 2-5 and Table 1.
- Steinhilber B, Haupt G, Miller R, Janssen P, Krauss I. Exercise therapy in patients with hip osteoarthritis: Effect on hip muscle strength and safety aspects of exercise-results of a randomized controlled trial. Mod Rheumatol. 2017;27(3):494 and Figure 1.
- Haupt G, Janßen P, Krauß I, Steinhilber B. Das Tübinger Hüftkonzept. In. Vol 1. Auflage. Essen: Verlag hellblau; 2014:1-176. https://verlag-hellblau.de/portfolio-item/tuebinger-hueftkonzept/

5. Who provided:

- Group sessions were introduced by one therapist. He is a physiotherapist and sports scientist with more than 20 years of experience in guiding hip patients and hip sports groups.

6. How

- Group (1/week, 60-90 minutes) with a group size of maximum 15 people.
- Home training sessions (2/week, 30-40 minutes each).

7. Where

- Group sessions took place in a gymnastics hall (ca. 70 m²) of the Dept. of Sports Medicine, the theory units in its lecture hall.

8. When and How much

- See No. 4

9. Tailoring

- Tailoring was personalized in terms of training dosage (Borg 6-7, clean movement execution for strength and balance tasks).
- From week 9 onwards, the participants had the opportunity to vary some of the exercises according to their preferences.

10. Modification

- Not applicable.

11. How well planned:

- Adherence and fidelity was assessed as displayed on page 4 and 5 of the main document. Adherence to group sessions was monitored by the supervisor, adherence to home training sessions by the subjects themselves.
- The participants were encouraged to inform the trainer in advance in case of absence from the group training. In these cases, the home training program was provided beforehand. Additional measures for therapy adherence were not necessary due to the very high compliance.

11. How well actual

- Details on adherence and fidelity are displayed in Table V of the main document.
