# Supplementary material for: Secondary Analysis of a Study on Exercise Therapy in Hip Osteoarthritis: Follow-Up Data on Pain and Physical Functioning
Source: Int J Environ Res Public Health. 2021 Aug 7;18(16):8366. doi: 10.3390/ijerph18168366 (PMC8393441; doi:10.3390/ijerph18168366)
Supplement: Supplementary file 1 [file ijerph-18-08366-s001.zip › ijerph-1279695-supplementary/ijerph-1279695-supplementary final/Roesel_Supplement_4_Treatment_satisfaction.pdf]

Supplement 4. Treatment satisfaction and recommendation (*n* = 112).

	Treatment sequence (n)	Intervention period	applicable	likely applicable	neither nor	likely inapplicable	not applicable	missing
**Satisfaction with the exercise intervention n [%]**	**E**-C (48)	t0-t3	42 (87.5%)	4 (8.3%)	1 (2.1%)	1 (2.1%)	0 (0%)	1
	C-**E** (32)	t3-t6	25 (78.1%)	4 (12.5%)	2 (6.3%)	1 (3.1%)	0 (0%)	1
	P-**E** (32)	t3-t6	27 (84.4%)	4 (12.5%)	1 (3.1%)	0 (0%)	0 (0%)	1
	**Total** (112)		94 (83.9%)	12 (10.7%)	4 (3.6%)	2 (1.8%)	0 (0%)	3
**Recommendation of the exercise intervention n [%]**	**E**-C (48)	t0-t3	45 (93.8%)	2 (4.2%)	0 (0%)	1 (2.1%)	0 (0%)	1
	C-**E** (32)	t3-t6	28 (87.5%)	3 (9.4%)	0 (0%)	1 (3.1%)	0 (0%)	1
	P-**E** (32)	t3-t6	27 (84.4%)	4 (12.5%)	0 (0%)	1 (3.1%)	0 (0%)	1
	**Total** (112)		100 (89.3%)	9 (8.0%)	0 (0%)	3 (2.7%)	0 (0%)	3

Footnote: Bold letters indicate the intervention phase to which satisfaction and recommendation is related to.
