# Supplementary material for: Secondary Analysis of a Study on Exercise Therapy in Hip Osteoarthritis: Follow-Up Data on Pain and Physical Functioning
Source: Int J Environ Res Public Health. 2021 Aug 7;18(16):8366. doi: 10.3390/ijerph18168366 (PMC8393441; doi:10.3390/ijerph18168366)
Supplement: Supplementary file 1 [file ijerph-18-08366-s001.zip › ijerph-1279695-supplementary/ijerph-1279695-supplementary final/Roesel_Supplement_5_Effect sizes_vs_t0_2021_revised.pdf]

**Supplement 5**: Mean (SD), change scores$, effect sizes (Hedges $g_z$ [12]) and 95%-CIs for clinical outcome measures (n = 137).

| | E-C | | | C-E | | | P-E | | |
|---|---|---|---|---|---|---|---|---|---|
| | *n* | *Mean (SD)* | *change score (ES; 95-CI)* | *n* | *Mean (SD)* | *change score (ES; 95-CI)* | *n* | *Mean (SD)* | *change score (ES; 95-CI)* |
| **SF36 bodily pain** | | | | | | | | | |
| t0 | 57 | 59.3 (16.0) | | 43 | 58.2 (19.2) | | 37 | 57.0 (19.7) | |
| t3 | 57 | 65.9 (18.5) | 6.80* (**0.36**; [0.09, 0.63]) | 43 | 57.0 (19.4) | -1.63 (-0.07; [-0.33, 0.27]) | 37 | 58.1 (19.2) | 1.64 (0.06; [-0.26, 0.38]) |
| t6 | 54 | 64.1 (21.5) | 6.35 (0.26; [-0.01, 0.53]) | 43 | 64.1 (21.5) | 7.25 (0.27; [-0.03, 0.58]) | 36 | 60.5 (18.6) | 5.37 (0.22; [-0.11, 0.55]) |
| t12 | 49 | 67.1 (19.2) | 6.86* (**0.39**; [0.10, 0.68]) | 32 | 60.3 (22.0) | 3.03 (0.15; [-0.20, 0.49]) | 34 | 60.8 (20.2) | 4.21 (0.21; [-0.14, 0.55]) |
| **WOMAC pain** | | | | | | | | | |
| t0 | 57 | 26.0 (15.2) | | 43 | 27.1 (16.9) | | 37 | 26.5 (16.5) | |
| t3 | 57 | 16.8 (14.2) | -9.16* (**0.62**; [0.33, 0.90]) | 43 | 26.7 (19.6) | -0.47 (0.03; [-0.27, 0.33]) | 37 | 22.9 (14.2) | -3.62 (0.24; [-0.09, 0.57]) |
| t6 | 54 | 18.9 (15.9) | -7.15* (**0.44**; [0.16, 0.72]) | 43 | 23.9 (19.8) | -3.21 (0.16; [0.14, 0.46]) | 36 | 20.1 (15.4) | -6.83* (**0.35**; [0.01, 0.68]) |
| t12 | 49 | 16.9 (13.4) | -8.08* (**0.56**; [0.25, 0.86]) | 33 | 21.9 (15.7) | -7.09* (**0.37**; [0.02, 0.72]) | 34 | 21.6 (15.7) | -5.94* (**0.33**; [0.02, 0.68]) |
| **WOMAC function** | | | | | | | | | |
| t0 | 57 | 24.7 (16.9) | | 43 | 25.5 (15.2) | | 37 | 27.2 (15.1) | |
| t3 | 57 | 15.6 (14.0) | -9.07* (**0.64**; [0.36, 0.93]) | 43 | 23.8 (16.1) | -1.73 (0.13; [-0.17, 0.43]) | 37 | 21.6 (13.7) | -5.60* (**0.43**; [0.09, 0.77]) |
| t6 | 54 | 16.7 (15.5) | -8.71* (**0.54**; [0.25, 0.82]) | 43 | 19.4 (14.1) | -6.14* (**0.38**; [0.07, 0.69]) | 36 | 21.9 (13.4) | -5.44* (**0.38**; [0.04, 0.72]) |
| t12 | 49 | 14.9 (13.8) | -9.03* (**0.59**; [0.28, 0.89]) | 33 | 20.6 (14.0) | -6.86* (**0.47**; [0.11, 0.83]) | 34 | 22.2 (15.4) | -5.22 (0.32; [-0.03, 0.67]) |
| **WOMAC stiffness** | | | | | | | | | |
| t0 | 57 | 28.9 (18.8) | | 43 | 36.1 (23.1) | | 37 | 37.3 (20.8) | |
| t3 | 57 | 20.2 (17.7) | -8.78* (**0.41**; [0.14, 0.68]) | 43 | 27.2 (19.5) | -8.84* (**0.50**; [0.18, 0.81]) | 37 | 28.5 (16.7) | -8.78* (**0.46**; [0.12, 0.79]) |
| t6 | 54 | 22.6 (17.4) | -7.13* (**0.33**; [0.06, 0.60]) | 43 | 27.3 (18.1) | -8.72*(**0.49**; [0.17, 0.80]) | 36 | 26.7 (16.4) | -11.1* (**0.55**; [0.20, 0.89]) |
| t12 | 49 | 24.0 (17.9) | -0.94 (0.26; [-0.26, 0.54]) | 33 | 23.0 (16.4) | -13.3* (**0.54**; [0.18, 0.90]) | 34 | 27.6 (18.5) | -9.85*(**0.52**; [0.16, 0.87]) |

$ Change from baseline within-group means

Positive effect sizes indicate benefit; ES in bold indicate CI does not contain 0.

* p<0.05. Null hypothesis: difference (mean at follow-up minus mean at baseline)=0.
