# Supplementary material for: Secondary Analysis of a Study on Exercise Therapy in Hip Osteoarthritis: Follow-Up Data on Pain and Physical Functioning
Source: Int J Environ Res Public Health. 2021 Aug 7;18(16):8366. doi: 10.3390/ijerph18168366 (PMC8393441; doi:10.3390/ijerph18168366)
Supplement: Supplementary file 1 [file ijerph-18-08366-s001.zip › ijerph-1279695-supplementary/ijerph-1279695-supplementary final/Roesel_Supplement_6_Effect sizes.pdf]

**Supplement 6**: Effect sizes (Hedges $g_z$ [13]) and 95%-CIs for clinical outcome measures (*n* = 137) in the voluntary exercise intervention period.

| | **Exercise intervention period of P-E, C-E** *(ES; 95%-CI)* |
|---|---|
| **C-E (n=43)** | **t6-t3** |
| SF36 bodily pain | **0.40 [0.09, 0.71]** |
| WOMAC pain | 0.13 [-0.17, 0.43] |
| WOMAC physical functioning | 0.27 [-0.03, 0.58] |
| WOMAC stiffness | -0.01 [-0.30, 0.29] |
| **P-E (n=37)** | **t6-t3** |
| SF36 bodily pain | 0.19 [-0.14, 0.52] |
| WOMAC pain | 0.23 [-0.10, 0.56] |
| WOMAC physical functioning | 0.01 [-0.32, 0.34] |
| WOMAC stiffness | 0.16 [-0.18, 0.48] |

Positive effect sizes indicate benefit.
