# Supplementary material for: Secondary Analysis of a Study on Exercise Therapy in Hip Osteoarthritis: Follow-Up Data on Pain and Physical Functioning
Source: Int J Environ Res Public Health. 2021 Aug 7;18(16):8366. doi: 10.3390/ijerph18168366 (PMC8393441; doi:10.3390/ijerph18168366)
Supplement: Supplementary file 1 [file ijerph-18-08366-s001.zip › ijerph-1279695-supplementary/ijerph-1279695-supplementary final/Roesel_Supplement_7_ANCOVA_137_2021_revised.pdf]

Supplement 7: ANCOVA for exercise intervention phase adjusted for baseline (*n* = 137)

	p-val	E-C (n=57)	C-E (n=43)	P-E (n=37)	Difference C-E - E-C**	p	Difference P-E - E-C**	p
**SF36 bodily pain**								
pre		59.3 (16.0)	57.0 (19.4)	58.1 (19.2)				
post		65.9 (18.5)	64.1 (21.5)	6.5 (18.6)				
ANCOVA	0.419	65.1* (2.12)	64.7 (2.44)	60.9 (2.66)	-0.41 (-6.80; 6.00)	0.900	-4.26 (-11.00; 2.47)	0.213
**WOMAC pain**								
pre		21.9 (15.2)	26.6 (19.6)	22.9 (14.7)				
post		16.8 (14.1)	23.9 (19.8)	20.1 (15.4)				
ANCOVA	0.058	16.6* (1.90)	23.3 (2.18)	21.1 (2.39)	**6.76** (1.04; 12.48)	**0.021**	4.52 (-1.52; 10.56)	0.141
**WOMAC function**								
pre		24.7 (16.9)	23.8 (16.1)	21.6 (13.7)				
post		15.6 (14.0)	19.4 (14.1)	21.9 (13.4)				
ANCOVA	**0.005**	15.0* (1.47)	19.3 (1.70)	22.8 (1.86)	4.25 (-0.195; 8.698)	0.061	**7.79** (3.09; 12.49)	**0.001**
**WOMAC stiffness**								
pre		28.9 (18.8)	27.2 (19.5)	28.5 (16.6)				
post		20.2 (17.7)	27.3 (18.1)	26.7 (16.4)				
ANCOVA	**0.026**	19.9* (2.05)	27.9 (2.36)	26.4 (2.60)	**7.94** (1.75, 14.12)	0.120	-1.42 (-8.33, 5.50)	0.686

*adjusted means (standard error)

** difference between adjusted means (95% CI)
