# Supplementary figures and images for: Secondary Analysis of a Study on Exercise Therapy in Hip Osteoarthritis: Follow-Up Data on Pain and Physical Functioning
Source: Int J Environ Res Public Health. 2021 Aug 7;18(16):8366. doi: 10.3390/ijerph18168366 (PMC8393441; doi:10.3390/ijerph18168366)

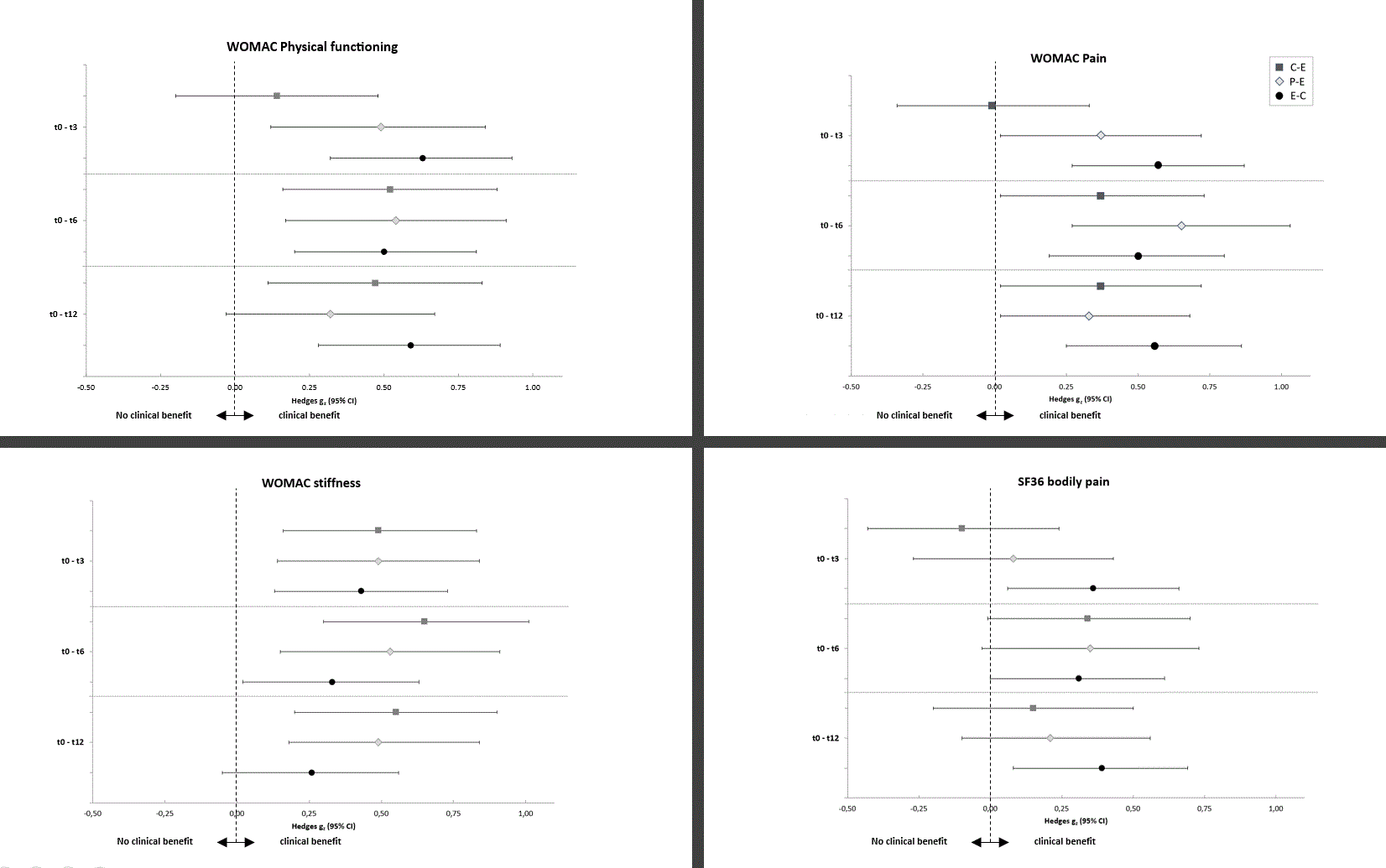

Supplement: Supplementary file 1 [file ijerph-18-08366-s001.zip › ijerph-1279695-supplementary/ijerph-1279695-supplementary final/Roesel_Supplement_3_ForestPlots.GIF]
